# Editorial: New insights into mechanisms of epigenetic modifiers in plant growth and development, volume II

**DOI:** 10.3389/fpls.2023.1213511

**Published:** 2023-06-20

**Authors:** Ming Luo, Tomasz Jacek Sarnowski, Marc Libault, Gabino Ríos, Jean-Benoit Charron, Nitin Mantri, Shoudong Zhang

**Affiliations:** ^1^ Key Laboratory of South China Agricultural Plant Molecular Analysis and Genetic Improvement, Guangdong Provincial Key Laboratory of Applied Botany, South China Botanical Garden, Chinese Academy of Sciences, Guangzhou, China; ^2^ Institute of Biochemistry and Biophysics Polish Academy of Sciences, Warsaw, Poland; ^3^ Department of Agronomy and Horticulture, Center for Plant Science Innovation, University of Nebraska, Lincoln, NE, United States; ^4^ Department of Citriculture and Plant Production, Valencian Institute for Agricultural Research (IVIA), Valencia, Spain; ^5^ Department of Plant Science, McGill University, Sainte-Anne-de-Bellevue, QC, Canada; ^6^ The Pangenomics Lab, School of Science, RMIT University, Bundoora, VIC, Australia; ^7^ The UWA Institute of Agriculture, The University of Western Australia, Perth, WA, Australia; ^8^ School of Agriculture, Yunnan University, Kunming, China

**Keywords:** epigenetic, histone modification, DNA methyaltion, RNA, plant growth

As we have learned, chromatin modifications, including histone modifications and DNA methylation, play a key role in plant development (Ng and Bird, 1999). However, accumulated evidence shows that, besides chromatin biochemical modifications, other epigenetic regulations such as chromatin architecture also function at a pivot point to regulate plant development (Zhang et al., 2021). In this Research Topic, five research papers describe multiple developmental facets mediated by different epigenetic mechanisms besides histone modifications. These manuscripts report experimental evidence or summarize recent advances in epigenetic regulations of some important developmental genes or development-related mechanisms. This Research Topic allows readers to learn of the latest advances in epigenetic regulations on seed germination, flowering time control, miRNA biogenesis and stability, secondary meristem maintenance as well as histone deacetylase 9 mediated day-length dependent hypocotyl cell elongation.

Flowering locus C(FLC)is a major determinant of flowering in Arabidopsis. Whereas the repression of *FLC* expression by autonomous pathway genes includes histone modifications, recent advances indicate that this process is much more complex. It has been shown that the precise control of *FLC* expression additionally involves chromatin architecture, RNA polymerase pausing, and ncRNA-mediated gene silencing. The review by Kyung et al. discusses how these novel mechanisms coupled with histone modifications may lead to the repression of *FLC* expression and provides the reader with a comprehensive review of autonomous pathway gene-mediated *FLC* repression *via* epigenetic regulations.

Since the discovery of miRNAs in *C. elegans*, they have been tightly connected to organisms’ development. Although there are different biogenesis mechanisms between plants and other organisms, miRNAs have been confirmed to play a key role in plant development, e.g., miRNA156/172 regulating developmental timing. For instance, pre-miRNAs that are transcribed from *MIR* genes by DNA-dependent RNA polymerase mediate *MIR* promoter accessibility and *MIR* gene transcription. In addition to this role, epigenetic factors also regulate miRNA biogenesis and abundance. Recent evidence has shown that a key component Serrate (SE) of miRNA processing complex can directly interact with CHROMATIN REMODELLER 2 (CHR2) and unwind pre-miRNA structure, thus preventing miRNA biogenesis and accumulation. However, CHR2 also can function as a positive regulator of *MIR* gene transcription *via* its chromatin remodeling activity. The different and even opposite roles of CHR2 in miRNA biogenesis embody the complexity of epigenetic regulations. This and other detailed advances in miRNA biogenesis and stability can be found in the review article by Zhang et al..

Secondary growth mediated by secondary meristems is crucial for plant radial thickening and plant axes strengthening. Whereas vascular cambium leads to secondary xylem and phloem, cork cambium or phellogen produce the periderm, with an important role to protect plants from insects, diseases, and the harmful effects of climate change. Secondary growth mediated by vascular cambium such as during xylogenesis, dormancy-activation periods of cambium, and secondary tissue regeneration after injury are under epigenetic regulations, involving histone modifications (H3K4me3), DNA methylation, chromatin remodeling, and miRNA-mediated DNA methylation. During periderm formation, phellogen-mediated cell division, differentiation, and regeneration are regulated by various epigenetic modifications, including increased DNA methylation followed by chromatin condensation, and H3K4me3 enrichment to activate genes involved in secondary cell wall deposition and programmed cell death. In addition, miRNAs targeting histone modifiers cause alterations in the histone modification landscapes and also mediate periderm differentiation and formation. More in-detailed epigenetic effects on plant secondary growth can be found in the review paper by Inácio et al..

Histone modifications play a key role in regulating developmental genes, and RPD3-like histone deacetylases, e.g., HDA6, HDA9, HDA19, etc., can form conserved SIN3-type histone deacetylase complexes to regulate plant responses to stresses and developmental cues. Among them, HDA6 maintains heterochromatin status by preventing DNA demethylation at heterochromatin regions through deacetylating H3K18ac, a crucial mark for DNA demethylases (Wang et al., 2021). Although HDA6, HDA9, and HDA19 are commonly involved in the regulation of Arabidopsis flowering time, they have different molecular targets; HDA6 represses *FLC* expression, HDA9 targets *AGL19*, and HDA19 regulates photoperiod genes. In the Research Topic, Lee et al. show that HDA9 represses *GIGANTEA* expression under short-day conditions, thus stimulating hypocotyl cell elongation.

Seed dormancy is not only an important developmental process but also affects plant survival and adaptation to adverse habitats. *Delay of germination 1* (*DOG1*) has been described as controlling seed dormancy by converging with the ABA signaling pathway to tightly repress seed germination. Previous studies with different Arabidopsis ecotypes adapted to summer (Bur) and winter (Cvi) seasons found that histone modification H3K4me3 remains stable during dormancy, and as dormancy declines, H3K4me3 level decreases. During the release of dormancy, H3K27me3 repressive mark slowly accumulated along *DOG1*. In this Research Topic, Han et al. show that HD2A and HD2B are recruited by HSI2 and HSL1 to downregulate *DOG1* expression and to release seed dormancy. These results show that various epigenetic modifications coordinate together to make a fine tune for seed dormancy and germination.

## Concluding remarks

In the Research Topic, readers will find how histone modifications affect developmental gene expression and regulation, e.g., histone acetylation on the *DOG1* gene and HDA19-mediated repression of *GIGANTEA.* In addition, this Research Topic brings the latest epigenetic advances on periderm development as well as the chromatin architecture effects on *FLC* expression and epigenetic interaction with miRNA biogenesis and stability. Given the fact that histone deacetylases interact with epigenetic machinery, such as chromatin remodeling complexes and numerous transcription factors involved in important regulatory processes, the further exploration of this field may lead to the deciphering of not yet recognized precise regulatory mechanisms controlling gene expression in the context of the response to changing environmental conditions. Thus, the articles presented in this Research Topic provide qualified and valuable knowledge for the epigenetic community.

## Author contributions

All authors listed have made a substantial, direct, and intellectual contribution to the work, and approved it for publication.
